# Supply and advertising of food and beverages with front-of-package warning labeling in schools of Metropolitan Lima 2023

**DOI:** 10.17843/rpmesp.2026.431.15249

**Published:** 2026-03-02

**Authors:** Mayra Meza-Hernández, Kiomi Yabiku-Soto, Francisco Diez-Canseco

**Affiliations:** 1 CRONICAS Center of Excellence in Chronic Diseases, Universidad Peruana Cayetano Heredia. Lima, Peru.

**Keywords:** Food Supply, Food Labeling, Food Advertising, Academic Institutions, Peru

## Abstract

**Objectives.:**

To describe the supply and advertising of food and beverages with front-of-package warning labeling in kiosks and cafeterias, and in school environments of public and private schools in Metropolitan Lima.

**Materials and methods.:**

A cross-sectional study was conducted in 45 schools in Metropolitan Lima between March and May 2023. The supply and advertising of food and beverages were observed in school kiosks during the school day and among street vendors located at the school exit gates.

**Results.:**

A total of 57 school kiosks and cafeterias from 45 schools were analyzed. Processed and ultra-processed foods and beverages were observed in 96.5% of them, and at least one product with front-of-package warning labeling (octagon) was identified in 89.5%. Street vendors were found at 41 schools, totaling 148 vendors. Processed and ultra-processed food and beverages were observed in 77.7% of them, and at least one food or beverage with an octagon was observed in 48.6%. Advertising for processed and ultra-processed foods was observed in 57.9% of school kiosks and cafeterias and in 27.7% of street vendors.

**Conclusions.:**

Evidence shows the exposure of schoolchildren to unhealthy food environments and non-compliance with regulations prohibiting the sale of foods with octagons both inside and around schools. It is necessary to reinforce information, awareness, and monitoring of the educational community to ensure the effectiveness of these policies in protecting the health of schoolchildren.

## INTRODUCTION

Overweight and obesity in children and adolescents is a public health problem in Latin America and the Caribbean ^1^. In Peru, as of 2023, two out of every five children between 6 and 13 years old, and three out of every ten adolescents between 12 and 17 years old, are overweight or obese ^2^. Unhealthy dietary habits and physical inactivity are the main risk factors to which children and adolescents are exposed ^3^. Unhealthy dietary habits include the increased consumption of ultra-processed beverages and foods, which are characterized as being highly caloric industrial formulations with low nutritional density ^4^. Furthermore, these beverages and foods often direct their advertising strategies toward children and adolescents, who are highly vulnerable to them as well as to exposure to the availability of these foods ^5^.

Frequent exposure of children and adolescents to the availability and advertising of ultra-processed products can influence their choice of these types of beverages and foods and increase their consumption ^6^. The school is a space where children and adolescents (schoolchildren hereafter) spend several hours of the day most of the year; therefore, the school food environment, which encompasses both the interior and the surroundings of the school, is a conducive environment to promote and foster healthy dietary habits that will persist throughout life ^7^. In this regard, the Food and Agriculture Organization of the United Nations (FAO) indicates that a healthy school food environment should encourage food choices compatible with a healthy diet that improves the nutrition of schoolchildren ^8^.

In Peru, in February 2019, the Ministry of Health (MINSA) published the "Guidelines for the promotion and protection of healthy eating in public and private educational institutions of regular basic education" (Guidelines hereafter) ^9^, which indicate the characteristics of the beverages and foods that can be offered in school kiosks and cafeterias. These Guidelines were published within the framework of the implementation of the "Law for the promotion of healthy eating for children and adolescents" (Law No. 30021) ^10^, whose manual establishes the parameters for a packaged beverage or food to be considered high in sugar, saturated fats, sodium, or containing trans fats and, if so, to include a front-of-package warning label (a black octagon) on its label to inform citizens of this content ^11^^,^^12^. The Guidelines highlight that school kiosks and cafeterias may only offer packaged beverages and foods that do not exceed these parameters. However, an exploratory study conducted in 15 schools in Metropolitan Lima in November 2019, four months after the introduction of the warnings, found that 7 out of 10 schools offered beverages or foods with octagons ^13^.

Evidence exists demonstrating that the food supply inside and outside schools is associated with the nutritional status of schoolchildren ^14^^,^^15^. Regarding the latter, in 2021, the Metropolitan Municipality of Lima enacted the "Ordinance promoting the generation of healthy environments for children and adolescents in Metropolitan Lima" (ORDINANCE No. 2366-2021), which prohibits the street vending of unhealthy foods such as candies, sugary drinks, ultra-processed products, and similar items within a 200-meter perimeter around schools ^16^. In the same exploratory study conducted at the end of 2019, prior to this ordinance, street vendors were observed at the exit gates of all evaluated schools ^13^.

In this context, it is relevant to evaluate compliance with regulations related to school food environments, four years after the publication of the Guidelines regulating the offer of beverages and foods in school kiosks and cafeterias and two years after the publication of the municipal ordinance regulating street vending around schools. Therefore, the present study seeks to describe the offer and advertising of beverages and foods in school kiosks and cafeterias and in the environments of public and private schools in Metropolitan Lima.

**KEY MESSAGES**

Motivation for the study. Overweight and obesity in children and adolescents is a public health problem; unhealthy dietary habits are one of the main risk factors.

Main findings. Evidence shows the exposure of schoolchildren to unhealthy food environments and non-compliance with regulations prohibiting the sale of foods with octagonal warning labels inside and around schools.

Implications. It is necessary to strengthen information, awareness, and monitoring of educational environments to guarantee healthy dietary habits.

## MATERIALS AND METHODS

### Design

An observational, descriptive, and cross-sectional study was conducted. Information on the offer and advertising of beverages and foods in school kiosks and cafeterias, as well as among street vendors outside the school, was collected between March and May 2023.

### Sample

Forty-five schools located in 20 districts of Metropolitan Lima, the capital of Peru, were selected through convenience sampling. The schools were selected based on a contact network of the research team formed by teachers and mothers with access to schools. Regular basic education schools, both public and private, were included, and alternative basic education schools, special basic education schools, technical-productive education schools, schools teaching only the initial level, and schools without a kiosk or cafeteria were excluded. In the included schools, the presence of street vendors located along the sidewalk where the school exit gate is located, as well as on the opposite sidewalk, was observed.

In each kiosk and cafeteria of the schools included in the sample, as well as in each street vending post, the food supply, ultra-processed products with front-of-package warning labels, and the presence of advertising for ultra-processed products were observed and recorded.

### Data Collection

Data were collected through non-participant observation. In the school kiosks and cafeterias located inside the schools, the observation was carried out by the teacher or mother contacted and duly trained by the research team through virtual meetings lasting an average of 60 minutes. The observations were conducted unannounced to avoid modifications in the actual food supply at the time of registration. Observation inside the schools was carried out during the school day, while the observation of street vendors was conducted by a member of the research team during the schoolchildren's dismissal time. In schools with differentiated dismissal schedules for each level taught, the observation was performed during the dismissal of the primary and secondary levels. This same methodology was successfully employed in the 2019 exploratory study ^13^.

Two observation sheets were used to record the observations: one for school kiosks and cafeterias and another for street vendors. Both were divided into two sections: the first recorded the offer of beverages and foods, and the second, the presence of advertising for processed products. The beverages and foods considered in both sheets were presented in three groups: (i) processed and ultra-processed beverages and foods, (ii) homemade preparations, and (iii) natural foods. These groups were in turn divided into 16 categories based on the Guidelines enacted by MINSA. The description of each category considered in the observation sheet is detailed in Table 1. In the categories corresponding to processed and ultra-processed beverages and foods, it was recorded whether at least one product with an octagon was observed. In the section corresponding to advertising, its presence and the form in which it was presented were recorded: (i) posters or signs, (ii) advertising attached to the furniture of the kiosk or street vendors' modules, and (iii) other types of advertising.

**Table 1:** Detail of beverage and food groups and categories.

Food and beverage groups		Definitions	Examples
Processed and ultra-processed foods and beverages			
	Bakery products	Sweet or savory foods made from wheat flour, packaged and branded	Sweet, savory, and filled cookies/biscuits, sponge cakes, cakes
	Sweets/Confectionery	Food products made primarily from sugar	Candies, chocolates, toffees
	Savory and sweet snacks	Fried, extruded, or baked savory snacks based on corn, cheese, among others	Potato chips, corn snacks, puffed wheat
	Packaged nuts and dried fruits	Nuts or seeds with added salt or sugar, packaged and branded	Sweet and salted peanuts, mixed nuts
	Beverages	Packaged and branded flavored and sweetened drinks	Sodas, soft drinks, juices, extracts
	Ice cream	Industrialized cream or water-based ice cream	Popsicles, ice cream bonbons
	Cereals	Packaged and branded breakfast cereals and cereal bars	Corn flakes, chocolate balls, colored rings
	Yogurt	Packaged and branded flavored and sweetened yogurt	Drinkable yogurt, stirred yogurt
	Instant soups	Packaged and branded instant soups	Soups in sachets or cups
	Others	Other packaged products that do not fit into the previous categories	-
Prepared foods and beverages			
	Sausage-based preparations	Preparations that include sausages as the main ingredient (industrialized meat products)	Burgers, fried nuggets, fried hot dogs, among others
	Homemade dish preparations	Homemade preparations frequently consumed in households	Arroz chaufa (fried rice), pollo broaster (breaded fried chicken), causa rellena (layered potato dish), among others
	Sandwiches without sausages	Sandwiches filled with different ingredients except sausages	Chicken, avocado, or cheese sandwiches, among others
	Natural beverages	Handcrafted drinks made from natural fruits or cereals	Soft drinks such as chicha morada, lemonade, 100% fruit juices, among others
	Refrigerated or frozen preparations	Handcrafted preparations that must be kept frozen or refrigerated	Frozen natural fruit soft drinks or juices, homemade gelatin, among others
	Others	Other prepared products that do not fit into the previous categories	-
Natural foods			
	Bulk nuts and dried fruits	Nuts and dried fruits sold in bulk with or without added salt or sugar	Peanuts, almonds, raisins
	Fruits and vegetables	Whole or chopped fruits or vegetables	Apple, banana, pineapple slices, peeled prickly pear, among others

### Data Analysis

The type of management and the location of the schools were obtained from the information provided by the contacts. The educational levels taught, as well as the number of students in each school, were obtained from the Educational Quality Statistics published by the Ministry of Education ^17^, while the socioeconomic level of each school's neighborhood (low, lower-middle, middle, upper-middle, and high) was based on the Stratified Maps of Metropolitan Lima at the block level according to household per capita income published by the National Institute of Statistics and Informatics in 2020 ^18^.

A univariate analysis of relative and absolute frequencies was performed to describe the characteristics of the schools, the offer of beverages and foods, the presence of octagons on beverages and foods, and the advertising in school kiosks and cafeterias as well as among street vendors. Stata version 17 statistical software was used for this analysis.

### Ethical Aspects

The study protocol was exempted from review by the Institutional Ethics Committee of the Universidad Peruana Cayetano Heredia, through certificate CIEI-321-30-23.

## RESULTS

Information from 57 school kiosks and cafeterias corresponding to 45 schools was analyzed, as 9 of them had more than one kiosk or cafeteria. The 45 schools are distributed across 20 districts in different areas of Metropolitan Lima: 37.8% in the south, 24.4% in the center, 20.0% in the north, and 17.8% in the east. 66.7% of the schools are publicly managed, and 68.9% are located in areas of middle and lower-middle socioeconomic levels. The median number of students per school is 1088 (Interquartile Range: 461 - 1451), and more than half of the schools (55.6%) have all three educational levels (initial, primary, and secondary). In 91.1% of the schools, street vendors of beverages and foods were found at the students' exit gate or on the opposite sidewalk (see details in Table 2).

**Table 2:** Characteristics of Metropolitan Lima schools included in the analysis (n=45).

Characteristics		n	%
Type of management			
	Public	30	66.7
	Private	15	33.3
Educational levels*			
	Primary	2	4.4
	Secondary	3	6.7
	Preschool and primary	5	11.1
	Primary and secondary	10	22.2
	Preschool, primary, and secondary	25	55.6
Location			
	Central Lima	11	24.4
	North Lima	9	20.0
	East Lima	8	17.8
	South Lima	17	37.8
Socioeconomic level*			
	High	5	11.1
	Upper middle	5	11.1
	Middle	17	37.8
	Lower middle	14	31.1
	Low	4	8.9
Median number of students*		1088 (461 - 1451)	
Median number of students according to number of educational levels*			
	One level	350 (322 - 448)	
	Two levels	1146 (622 - 1387)	
	Three levels	1175 (781 - 1488)	
Number of street vendors at the exit gate			
	No street vendors	4	8.9
	1 street vendor	6	13.3
	2 street vendors	7	15.6
	3 street vendors	9	20.0
	4 street vendors	6	13.3
	5 street vendors	6	13.3
	6 street vendors	3	6.7
	7 street vendors	3	6.7
	8 street vendors	1	2.2
Number of kiosks per educational institution			
	1 kiosk or cafeteria	36	80
	2 kiosks or cafeterias	6	13.3
	3 kiosks or cafeterias	3	6.7

*Information based on the Educational Quality Statistics (ESCALE) of the Ministry of Education of Peru. **The socioeconomic level is based on the 2020 Stratified Maps of Metropolitan Lima at the block level according to per capita household income of the National Institute of Statistics and Informatics (INEI).

### Offer of beverages and foods in school kiosks and cafeterias

Regarding the offer of beverages and foods, of the 57 school kiosks and cafeterias included in the analysis, processed and ultra-processed products were observed in 96.5%, and at least one product with an octagon was observed in 89.5%. The categories of processed and ultra-processed products found most frequently were bakery products (96.5%), industrialized beverages (89.5%), candies (86.0%), and savory and sweet snacks (84.2%). Of the total kiosks and cafeterias offering bakery products, at least one product with an octagon was observed in 80.0%; of those with savory and sweet snacks, in 79.2%; of those offering candies, in 69.4%; and of those offering industrialized beverages, in 58.8%. The proportion of kiosks and cafeterias offering products with octagons in other categories can be consulted in Table 3.

**Table 3:** Food and beverage offerings in school kiosks and cafeterias and from street vendors at the gates of the 45 schools in Metropolitan Lima.

Characteristics			School kiosks and cafeterias (n=57)		Street vendors at the school gate (n=148)	
			n	%	n	%
Offering of processed and ultra-processed foods and beverages						
	Bakery products		55	96.5	75	50.7
		With warning labels (octógonos)	44	80.0	49	65.3
	Sweets/Confectionery		49	86.0	64	43.2
		with warning labels (octógonos)	34	69.4	35	54.7
	Savory and sweet snacks		48	84.2	66	44.6
		with warning labels (octógonos)	38	79.2	49	74.2
	Packaged nuts and dried fruits		16	28.1	8	5.4
		with warning labels (octógonos)	6	37.5	5	62.5
	Beverages		51	89.5	62	41.9
		with warning labels (octógonos)	30	58.8	22	35.5
	Ice cream		16	28.1	29	19.6
		with warning labels (octógonos)	9	52.9	14	48.3
	Cereals		20	35,1	25	16.9
		with warning labels (octógonos)	13	65.0	22	88.0
	Yogurt		19	33.3	33	22.3
		with warning labels (octógonos)	8	42.1	9	27.3
	Instant soups		2	3.5	1	0.7
		with warning labels (octógonos)	2	100.0	1	100.0
	Others (ingredients)		2	3.5	1	0.7
		with warning labels (octógonos)	1	50.0	0	0.0
Offering of prepared foods and beverages						
	Sausage-based preparations		30	52.6	30	20.3
	Homemade dish preparations		34	59.7	30	20.3
	Sandwiches without sausages		32	56.1	19	12.8
	Natural beverages		32	56.1	33	22.3
	Refrigerated or frozen preparations		28	49.1	36	24.3
	Other preparations		3	5.3	14	9.5
Offering of natural foods						
	Bulk nuts and dried fruits		14	24.6	8	5.4
	Fruits and vegetables		29	50.9	21	14.2
Categories of processed and ultra-processed foods and beverages observed						
	No category		2	3.5	33	22.3
	1 category		0	0.0	47	31.8
	2 categories		2	3.5	6	4.1
	3 categories		2	3.5	7	4.7
	4 categories		17	29.8	19	12.8
	5 categories		17	29.8	17	11.5
	6 categories		10	17.5	13	8.8
	7 categorías		3	5.3	4	2.7
	8 categories		2	3.5	1	0.7
	9 categories		2	3.5	1	0.7
Categories of processed and ultra-processed foods and beverages with warning labels (octógonos) observed						
	No category		6	10.5	76	51.4
	1category		4	7.0	20	13.5
	2 categories		7	12.3	12	8.1
	3 categories		16	28.1	17	11.5
	4 categories		13	22.8	10	6.8
	5 categories		6	10.5	10	6.8
	6 categories		2	3.5	1	0.7
	7 categories		1	1.8	1	0.7
	8 categories		0	0.0	1	0.7
	9 categories		2	3.5	0	0.0

On the other hand, 86.0% of school kiosks and cafeterias offered homemade preparations. The categories of homemade preparations observed most frequently were homemade dish preparations (59.7%), sandwiches without cold cuts (56.1%), and beverages prepared with natural ingredients (56.1%). Furthermore, in 61.4% of school kiosks and cafeterias, the sale of natural foods was observed, such as bulk nuts (24.6%) and fruits and vegetables (50.9%) (Table 3).

### Offer of beverages and foods among street vendors

In the 41 schools with street vendors, a total of 148 vendors were observed. 77.7% of them offered processed and ultra-processed beverages and foods, and in 48.6%, at least one beverage or food with an octagon was observed. The most observed categories of processed and ultra-processed products were bakery products (50.7%), savory and sweet snacks (44.6%), candies (43.2%), and industrialized beverages (41.9%). Of the total street vendors offering savory and sweet snacks, at least one product with an octagon was observed in 74.2%; of those offering bakery products, in 65.3%; of those offering candies, in 54.7%; and of those offering industrialized beverages, in 35.5%. The proportion of street vendors offering products from other categories can be consulted in Table 3.

56.8% of street vendors offered homemade preparations. Refrigerated or frozen preparations (24.3%), beverages prepared with natural ingredients (22.3%), homemade dish preparations (20.3%), and cold cut-based preparations (20.3%) were the most observed categories. Regarding the offer of natural foods, it was observed that 17.6% of street vendors offered this type of product: fruits and vegetables were observed in 14.2%, and bulk nuts in 5.4% (Table 3).

### Advertising in school kiosks or cafeterias and in street vendor modules

57.9% of school kiosks and cafeterias and 27.7% of street vendors displayed some type of advertising for ultra-processed beverages and foods. Of the kiosks and cafeterias that displayed advertising, 87.9% had the advertising attached to the furniture and 42.4% in the form of posters or signs. Regarding the street vendors who presented advertising, 95.1% had it attached to their vending modules, 22% in the form of posters or signs, and 4.9% on their clothing. The number and type of advertising by establishment can be seen in Table 4.

**Table 4:** Presence and type of advertising for processed and ultra-processed foods and beverages in school kiosks and cafeterias and in street vendor stalls at the gates of 45 schools in Metropolitan Lima

Characteristics		School kiosks and cafeterias with advertising (n=33)		Street vendor stalls with advertising (n=41)	
		n	%	n	%
	Presence of advertising	33	57.9	41	27.7
Type of advertising*					
	Poster	14	42.4	9	22.0
	Furniture	29	87.9	39	95.1
	Other (clothing items)	0	0.0	2	4.9
Number of establishments with advertising attached to furniture					
	1 piece of furniture	8	27.6	22	56.4
	2 pieces of furniture	7	24.1	16	41.0
	3 pieces of furniture	9	31.0	0	0.0
	4 pieces of furniture	2	6.9	0	0.0
	5 pieces of furniture	1	3.4	0	0.0
	6 pieces of furniture	2	6.9	1	2.6
Number of establishments with advertising posters or signs					
	1 poster	3	21.4	4	44.4
	2 posters	2	14.3	2	22.2
	3 posters	5	35.7	0	0.0
	4 posters	2	14.3	0	0.0
	5 posters	2	14.3	1	11.1
	6 posters	0	0.0	1	11.1
	7 posters	0	0.0	1	11.1

*The sum of the values exceeds 100% since more than one type of advertising was observed in some establishments.

## DISCUSSION

The results show that nearly all of the 57 kiosks and cafeterias in 45 public and private schools in Metropolitan Lima offer processed and ultra-processed beverages and foods. Furthermore, four years after the publication of the Guidelines prohibiting the offer of products with octagons in schools ^9^, 9 out of 10 kiosks and cafeterias were selling processed and ultra-processed products high in sugar, sodium, or saturated fats, or with trans fats. Added to this, only 6 out of 10 kiosks and cafeterias offered homemade preparations, natural refreshments, or sandwiches without cold cuts, and only half of the kiosks and cafeterias offered fruits and/or vegetables. This provides evidence that schoolchildren are exposed to ultra-processed products at school.

The results are similar and in some cases more concerning than those found in 2019, four months after the Guidelines were published, when the 15 schools evaluated offered processed and ultra-processed products, and 73.3% offered products with octagons ^13^, compared to 90% in the current study.

The prohibition of the sale of unhealthy beverages and foods in schools is an effective strategy in the fight against the increase in childhood overweight ^19^; therefore, several Latin American countries, in addition to Peru, have implemented policies that restrict the offer of these foods in schools. Unfortunately, the deficient implementation of regulations governing the food supply in schools has also been observed in other countries in the region. In Mexico, 8 years after the publication of the directive prohibiting the sale of foods and beverages that do not meet the nutritional criteria established therein, only 1% of the 103 evaluated school kiosks fully complied with the regulations ^20^. In Ecuador, nine years after the publication of its policy regulating the sale of unhealthy foods in school kiosks, kiosk owners, school principals, and parents indicated that the policy is not complied with ^21^. Similarly, in Costa Rica, it was evidenced that 76.6% of foods sold in school kiosks did not meet the nutritional criteria required in the policy prohibiting the offer of products and preparations with a high content of fats, total sugars, and sodium in schools nine years after its publication ^22^.

In contrast to this majority situation, in Chile, it was evidenced that the prohibition of the sale of beverages and foods with octagons in school kiosks reduced the availability of these products from 90.4% to 15.0% two years after its publication ^23^. The Chilean experience reveals that school feeding policies can be effective if they are efficiently implemented.

In addition to the offer inside the schools, street vendors were observed outside the vast majority of evaluated schools, which demonstrates non-compliance with the 2021 municipal ordinance that restricts street vending within 200 meters of schools in Metropolitan Lima ^16^. Furthermore, half of the street vendors offered processed and ultra-processed beverages and foods with octagons, indicating that even if schools complied with prohibiting their sale, schoolchildren will continue to find these products at the exit gate. In 2019, a very similar situation was found regarding the presence of street vendors ^13^, suggesting that despite the enactment of the ordinance, no improvements have occurred.

It is worth noting that the food categories offered most frequently in kiosks and by street vendors were bakery products including industrialized cookies and cakes, snacks, candies or sweets, and industrialized beverages. A similar situation was found in Mexico, where bakery products, sweets, and sugary drinks such as sodas, nectars, and juices were found in more than 90% of evaluated school kiosks ^20^. This shows that despite the prohibition of the offer of these products, there is a high availability of them, which, added to their low price, increases schoolchildren's accessibility to these products.

Added to the high accessibility of ultra-processed beverages and foods, advertising for them was found in 6 out of 10 school kiosks or cafeterias, which influences schoolchildren's purchasing decisions ^24^, even more so if it is located within a space where they spend several hours of the day, such as the school. Although Peru does not have a regulation prohibiting advertising in the school environment, it is required that any advertising presenting a beverage or food with octagons must include the same in the upper right corner of the advertisement. Based on this requirement, the main type of advertising found in the present study is attached to furniture such as freezers, shelves, display cases, among others, since this type of advertising only includes the brand or name of beverages and foods easily identifiable by schoolchildren, but not the product itself, which means that, under current regulation, they are not required to comply with the requirement to include the corresponding octagons in said advertising.

Faced with the situation described in this study, it is relevant to reflect on the possible reasons for non-compliance with these policies, which could include deficient supervision by the responsible authorities ^20^^-^^22^, the lack of empowerment of the educational community to exercise a supervisory role, and the absence of effective sanctions for non-compliance with the policy ^21^. For proper implementation, policies must explicitly state the methods and those responsible for supervision, who should submit periodic reports of their visits. Likewise, corresponding sanctions for non-compliance with the policy must be detailed. On the other hand, the authorities responsible for implementing the policy must ensure that school kiosk and cafeteria owners, as well as those in charge of supervision, receive training that allows them to correctly interpret what is stipulated in the policy ^21^^,^^22^. Just as each section of the policy should include previously defined terms to ensure its correct and easy interpretation; for example, Ordinance No. 2366-2021 establishes a restricted zone for the street vending of unhealthy foods, however, it does not define which foods are considered unhealthy, which can generate confusion among vendors and consequently lead to policy non-compliance.

Another possible reason for the found situation relates to the dietary preferences of schoolchildren. In this regard, qualitative studies conducted with school kiosk owners report that healthy foods are unattractive to this population ^21^, which may be due to both deficient education on healthy eating and constant exposure to advertising for ultra-processed foods in various media ^20^^-^^22^. Furthermore, the presence of street vendors deepens this problem, as schoolchildren would seek to obtain unhealthy foods before or after classes outside the school ^20^^-^^22^. Additionally, it has been identified that in schoolchildren from low-income schools, the presence of the octagon on ultra-processed foods and beverages does not seem to be sufficient for choosing healthier foods ^25^. Furthermore, many of the processed and ultra-processed foods sold in schools are generally presented in small packaging (front face smaller than 50 cm^2^), which, according to the policy established in Peru, may be exempt from carrying octagons despite having a high content of one or more regulated nutrients; this can generate confusion, as schoolchildren may assume a food is healthy when it may not be ^26^.

Therefore, it is highly relevant, first, to establish an educational program on healthy eating directed at schoolchildren as well as their families and teachers to raise awareness about the possible consequences of consuming unhealthy foods. Second, supervision must be made effective both in school kiosks and cafeterias and among street vendors offering unhealthy foods around schools. Third, conduct advertising campaigns for healthy foods comparable to those that exist for unhealthy foods, in addition to generating policies that prohibit any type of advertising for processed and ultra-processed beverages and foods both inside and in the immediate environment of schools, thereby contributing to the implementation of healthy school food environments.

Finally, there would also be economic reasons, among which is the low purchasing power of schoolchildren ^20^^,^^22^, which does not allow them to acquire healthy foods that usually have higher prices than processed and ultra-processed foods ^27^. Furthermore, on the part of the kiosk owners, the need to cover expenses inherent to their commercial activity, such as rental costs, personnel, and basic services, is their main barrier to varying the food offer; since the offer of healthy food is unattractive to schoolchildren, it is not demanded in a similar way as unhealthy products, which translates into a decrease in their income and therefore low profitability ^21^^,^^22^.

The offer and advertising of ultra-processed products in school kiosks and cafeterias, as well as in school environments, can favor the adoption of unhealthy dietary habits in children and adolescents. To address this problem, it is necessary for health authorities to provide tools to school kiosk and cafeteria owners that allow them to offer healthy, attractive, and accessible foods, while ensuring their profitability. A concrete measure is the preparation of lists of recommended beverages, foods, and preparations, adapted to the different seasons of the year and incorporating local ingredients. Another strategy consists of implementing subsidies for healthy foods and applying taxes to unhealthy products, following the example of sugary drinks, whose regulation has demonstrated beneficial health effects ^28^^,^^29^. These policies can incentivize owners to offer healthy and profitable options more frequently. Finally, it is essential to review and modify the current regulations, as regulatory gaps persist, such as the lack of advertising restrictions in the school environment, the lack of precision regarding the mandatory use of front-of-package warning labels in advertisements showing the brand but not the product, and the lack of a clear definition of unhealthy foods in Municipal Ordinance No. 2366-2021. Collectively, these actions would allow for the strengthening of the school environment as a space that effectively promotes healthy eating.

A strength of the study is that teachers or mothers were used as observers, who recorded the offer and advertising to which schoolchildren are daily exposed within the school. This would have been complicated for individuals outside the institution, whose presence could induce a change in the offer of beverages and foods.

Among the limitations of the study is the lack of representativeness of the sample, which limits the generalization of the results. Nonetheless, the inclusion of nearly 50 public and private schools from different areas and socioeconomic levels offers valuable results on the food supply and compliance with current regulations. Another limitation is the heterogeneity of the observers; however, this was controlled through personalized and thorough training in data recording in the instrument. Likewise, it is considered a limitation that observations were conducted on a single date, so possible variations in the offer of beverages and foods could not be captured. Finally, the observations only recorded the presence of beverages and foods by categories, without determining the quantity or diversity of products offered in each category or the sales, which would provide more information on the foods offered.

In conclusion, in nearly all the school kiosks and cafeterias observed, processed and ultra-processed beverages and foods are offered, and in 9 out of 10, beverages and foods with octagons are offered, while fruits and/or vegetables are offered in only half. In 6 out of 10 kiosks and cafeterias, advertising for processed and ultra-processed beverages and foods was observed. Furthermore, the street vending of processed and ultra-processed beverages and foods with octagons is observed in more than half of the schools. This situation reflects that children and adolescents are exposed to unhealthy school food environments despite having current regulations governing this issue. It is fundamental that the authorities responsible for implementing the policies strengthen and guarantee the monitoring and surveillance of compliance with them to ensure their effectiveness and achieve their objectives of improving the public health of children and adolescents.
