# Supplementary material for: Supply and advertising of food and beverages with front-of-package warning labeling in schools of Metropolitan Lima 2023
Source: Rev Peru Med Exp Salud Publica. 2026 Mar 2;43(1):41–50. doi: 10.17843/rpmesp.2026.431.15249 (PMC13245979; doi:10.17843/rpmesp.2026.431.15249)
Supplement: Translation

# Translation: Oferta y publicidad de bebidas y alimentos con etiquetado frontal de advertencia en escuelas de Lima Metropolitana 2023

**Authors:** Mayra Meza-Hernández, Kiomi Yabiku-Soto, Francisco Diez-Canseco

**Affiliations:** 1 CRONICAS Centro de Excelencia en Enfermedades Crónicas, Universidad Peruana Cayetano Heredia, Lima, Perú.

**Keywords:** Abastecimiento de alimentos, Etiquetado de alimentos, Publicidad de alimentos, Instituciones Académicas, Perú

## Abstract

**Objetivos.:**

Describir la oferta y publicidad de bebidas y alimentos con etiquetado frontal de advertencia en quioscos y cafeterías y, en entornos escolares de escuelas públicas y privadas de Lima Metropolitana.

**Materiales y métodos.:**

Se realizó un estudio transversal en 45 escuelas de Lima Metropolitana entre marzo y mayo del 2023. Se observó la oferta y publicidad de bebidas y alimentos en los quioscos escolares durante la jornada escolar y en vendedores ambulantes ubicados en la puerta de salida de las escuelas.

**Resultados.:**

Se analizaron 57 quioscos y cafeterías escolares de 45 escuelas. En el 96,5% se observaron bebidas y alimentos procesados y ultraprocesados, y en el 89,5% se observó al menos una bebida o alimento con etiquetado frontal de advertencia (octógono). En 41 escuelas se hallaron vendedores ambulantes, sumando un total de 148 vendedores. En el 77,7% de ellos se observaron bebidas y alimentos procesados y ultraprocesados y en el 48,6% se observó al menos una bebida o alimento con octógono. Se observó publicidad de alimentos procesados y ultraprocesados en el 57,9% de quioscos y cafeterías escolares y en el 27,7% de vendedores ambulantes.

**Conclusiones.:**

Se evidencia la exposición de los escolares a entornos alimentarios no saludables y el incumplimiento de las regulaciones que prohíben la venta de alimentos con octógonos tanto dentro como alrededor de las escuelas. Se requiere reforzar la información, sensibilización y el monitoreo de la comunidad educativa para garantizar la efectividad de estas políticas en el cuidado de la salud de los escolares.

## INTRODUCCIÓN

El sobrepeso y la obesidad en niños, niñas y adolescentes es un problema de salud pública en América Latina y el Caribe ^1^. En Perú, al 2023, dos de cada cinco niños y niñas entre 6 y 13 años, y tres de cada diez adolescentes entre 12 y 17 años tienen sobrepeso u obesidad ^2^. Los hábitos alimentarios no saludables y la inactividad física son los principales factores de riesgo a los que se encuentran expuestos los niños, niñas y adolescentes ^3^. Los hábitos alimentarios no saludables incluyen el incremento del consumo de bebidas y alimentos ultraprocesados, que se caracterizan por ser formulaciones industriales altamente calóricas y de baja densidad nutricional ^4^. Además, estas bebidas y alimentos suelen dirigir sus estrategias publicitarias a niños, niñas y adolescentes, quienes son altamente vulnerables a las mismas así como a la exposición a la oferta de estos alimentos ^5^.

La frecuente exposición de niños, niñas y adolescentes a la oferta y publicidad de productos ultraprocesados puede influir su elección de este tipo de bebidas y alimentos e incrementar su consumo ^6^. La escuela es un espacio donde niños, niñas y adolescentes (*escolares* en adelante) pasan varias horas del día la mayor parte del año, por lo tanto, el entorno alimentario escolar, que comprende tanto el interior como los alrededores de la escuela, es un ambiente propicio para promover y fomentar hábitos alimentarios saludables que permanecerán a lo largo de la vida ^7^. Al respecto, la Organización de las Naciones Unidas para la Alimentación y la Agricultura (FAO) indica que un entorno alimentario escolar saludable debe propiciar la elección de alimentos compatibles con una alimentación saludable y que mejore la nutrición de los escolares ^8^.

En el Perú, en febrero del 2019, el Ministerio de Salud (MINSA) publicó los “Lineamientos para la promoción y protección de la alimentación saludable en las instituciones educativas públicas y privadas de la educación básica regular” (Lineamientos en adelante) ^9^, los cuales indican las características de las bebidas y alimentos que pueden ofertarse en quioscos y cafeterías escolares. Estos Lineamientos se publicaron en marco de la implementación de la “Ley de promoción de la alimentación saludable para niños, niñas y adolescentes” (Ley N° 30021) ^10^, en cuyo manual se establecen los parámetros para que una bebida o alimento envasado sea considerado alto en azúcar, grasas saturadas, sodio o que contiene grasas trans y, de ser así, incluya un etiquetado frontal de advertencia (un octógono negro) en su etiqueta, que informe de este contenido a los ciudadanos ^11^^,^^12^. Los *Lineamientos* resaltan que en quioscos y cafeterías escolares solo se pueden ofertar bebidas y alimentos envasados que no sobrepasen estos parámetros. Sin embargo, un estudio exploratorio realizado en 15 escuelas de Lima Metropolitana en noviembre del 2019, cuatro meses después de la introducción de las advertencias, encontró que 7 de cada 10 escuelas ofertaban bebidas o alimentos con octógonos ^13^.

Existe evidencia que demuestra que, la oferta de alimentos en el interior y el exterior de las escuelas se asocia al estado nutricional de los escolares ^14^^,^^15^. Sobre esto último, en el 2021, la Municipalidad Metropolitana de Lima promulgó la “Ordenanza que promueve la generación de entornos saludables para los niños, niñas y adolescentes en Lima Metropolitana” (ORDENANZA N° 2366-2021) la cual prohíbe el comercio ambulatorio de alimentos no saludables como golosinas, bebidas azucaradas, productos ultraprocesados y similares en el perímetro de 200 metros alrededor de las escuelas ^16^. En el mismo estudio exploratorio realizado a finales del 2019, previo a esta ordenanza, se observaron vendedores ambulantes en la puerta de salida de todas las escuelas evaluadas ^13^.

En este contexto, resulta relevante evaluar el cumplimiento de las normativas relacionadas a los entornos alimentarios escolares, a cuatro años de la publicación de los *Lineamientos* que regulan la oferta de bebidas y alimentos en quioscos y cafeterías escolares y a dos años de publicada la ordenanza municipal que regula el comercio ambulatorio alrededor de las escuelas. Por lo tanto, el presente estudio busca describir la oferta y publicidad de bebidas y alimentos en quioscos y cafeterías escolares y en los entornos de escuelas públicas y privadas en Lima Metropolitana.

**MENSAJE CLAVE**

Motivación para el estudio. El sobrepeso y la obesidad en niños, niñas y adolescentes es un problema de salud pública, los hábitos alimentarios no saludables es uno de los principales factores de riesgo.

Principales hallazgos. Se evidencia la exposición de los escolares a entornos alimentarios no saludables y el incumplimiento de las regulaciones que prohíben la venta de alimentos con octógonos dentro y alrededor de las escuelas.

Implicancias. Se requiere reforzar la información, sensibilización y el monitoreo de los entornos educativos para garantizar hábitos alimentarios saludables.

## MATERIALES Y MÉTODOS

### Diseño

Se realizó un estudio observacional, descriptivo y transversal. La información sobre la oferta y publicidad de bebidas y alimentos en quioscos y cafeterías escolares, así como entre los vendedores ambulantes a las afueras de la escuela, se recolectó entre marzo y mayo del 2023.

### Muestra

Se seleccionaron 45 escuelas ubicadas en 20 distritos de Lima Metropolitana, capital del Perú, mediante un muestreo por conveniencia. Las escuelas se seleccionaron en base a una red de contactos del equipo de investigación formada por docentes y madres de familia con acceso a escuelas. Se incluyeron escuelas de educación básica regular, tanto públicas como privadas, y excluyeron escuelas de educación básica alternativa, escuelas de educación básica especial, escuelas de educación técnico-productiva, escuelas que impartían solo el nivel inicial y escuelas sin un quiosco o cafetería. En las escuelas incluidas se observó la presencia de vendedores ambulantes ubicados a lo largo de la acera donde se encuentra la puerta de salida de la escuela, así como en la acera del frente.

En cada quiosco y cafetería, de las escuelas incluidas en la muestra, así como en cada puesto de venta ambulante, se observó y registró la oferta de alimentos, de productos ultraprocesados con etiquetado frontal de advertencia, y la presencia de publicidad de productos ultraprocesados.

### Recolección de datos

Los datos fueron recolectados mediante observación no participante. En los quioscos y cafeterías escolares ubicadas en el interior de las escuelas, la observación fue realizada por el docente o madre de familia contactado y debidamente capacitado por el equipo de investigación mediante reuniones virtuales de 60 minutos en promedio. Las observaciones se realizaron de manera inopinada, con el fin de evitar modificaciones en la oferta de alimentos real al momento del registro. La observación en el interior de las escuelas se realizó durante la jornada escolar, mientras que la observación de vendedores ambulantes fue llevada a cabo por un miembro del equipo de investigación durante la hora de salida de los escolares. En las escuelas con horarios de salida diferenciados por cada nivel impartido, la observación se efectuó durante la salida de los niveles de primaria y secundaria. Esta misma metodología fue empleada con éxito en el estudio exploratorio del 2019 ^13^.

Para el registro de las observaciones se emplearon dos fichas de observación: una para quioscos y cafeterías escolares y otra para vendedores ambulantes. Ambas se dividieron en dos secciones: en la primera se registró la oferta de bebidas y alimentos, y en la segunda, la presencia de publicidad de productos procesados. Las bebidas y alimentos considerados en ambas fichas se presentaron en tres grupos: (i) bebidas y alimentos procesados y ultraprocesados, (ii) preparaciones caseras y (iii) alimentos naturales. Estos grupos a su vez se dividieron en 16 categorías basadas en los Lineamientos promulgados por el MINSA. La descripción de cada categoría considerada en la ficha de observación se detalla en la Tabla 1. En las categorías correspondientes a las bebidas y alimentos procesados y ultraprocesados se registró si se observaba al menos un producto con octógono. En la sección correspondiente a la publicidad se registró su presencia y la forma en que se presenta: (i) afiches o carteles, (ii) publicidad adherida a los bienes muebles del quiosco o módulos de los vendedores ambulantes y (iii) otros tipos de publicidad.

**Tabla 1:** Detalle de los grupos y categorías de bebidas y alimentos.

Grupos de bebidas y alimentos		Definiciones	Ejemplos
Bebidas y alimentos procesados, y ultraprocesados			
	Productos de panadería	Alimentos dulces o salados elaborados con base en harina de trigo envasados y con marca	Galletas dulces, saladas y rellenas, bizcochos, queques
	Golosinas	Productos alimenticios elaborados principalmente con azúcar	Caramelos, chocolates, *toffees*
	Snacks salados y dulces	Bocaditos salados fritos, extruidos u horneados en base a maíz, queso entre otros	Chips de papas fritas, snacks de maíz, trigo atomico
	Frutos secos envasados	Nueces o semillas con sal o azúcar añadida envasados y con marca	Maní salado y dulce, mix de frutos secos
	Bebidas	Bebidas saborizadas y endulzadas, envasadas y con marca	Gaseosas, refrescos, jugos, extractos
	Helados	Helados industrializados de crema o hielo	Paletas, bombones de helado
	Cereales	Cereales para desayuno y barras de cereal envasados y con marca	Hojuelas de maíz, bolitas de chocolate, aritos de colores
	Yogurt	Yogur saborizado y endulzado, envasados y con marca	Yogurt bebible, yogurt batido
	Sopas instantáneas	Sopas instantáneas envasadas y con marca	Sopas en sobre o en vaso
	Otros	Otro producto envasado que no encaje en las categorías anteriores	-
Bebidas y alimentos preparados			
	Preparaciones en base a embutidos	Preparaciones que incluyan como insumo principal embutidos (Productos cárnicos industrializados)	Pan con hamburguesa, *nuggets* fritos, *hot dogs* fritos, entre otros.
	Preparaciones de platos caseros	Preparaciones caseras frecuentemente consumidas en los hogares	Arroz chaufa, pollo broaster, causa rellena entre otros.
	Sánguches sin embutidos	Sandwich rellenos de diferentes insumos excepto los embutidos	Sandwiches de pollo, palta, queso, entre otros
	Bebidas naturales	Bebidas elaboradas artesanalmente en base a futas o cereales naturales	Refrescos como chicha morada, limonada, jugos de 100% frutas, entre otros.
	Preparaciones refrigeradas o congeladas	Preparaciones artesanales que deben mantenerse congeladas o refrigeradas	Refrescos o jugos de fruta natural congelados, gelatina casera, entre otros.
	Otras	Otro producto preparado que no encaje en las categorías anteriores	-
Alimentos naturales			
	Frutos secos a granel	Frutos secos expendidos a granel con o sin sal ni azúcar añadida	Maní, almendras, pasas
	Frutas y verduras	Frutas o verduras enteras o picadas	Manzana, plátano, rodajas de piña, tuna pelada, entre otros

### Análisis de datos

El tipo de gestión y la ubicación de las escuelas se obtuvieron de la información brindada por los contactos. Los niveles educativos impartidos, así como el número de alumnos en cada escuela, se obtuvieron de la Estadística de la Calidad Educativa publicada por el Ministerio de Educación ^17^, mientras que el nivel socioeconómico del barrio de cada escuela (bajo, medio bajo, medio, medio alto y alto) se basó en los Planos estratificados de Lima Metropolitana a nivel de manzanas según ingreso per cápita del hogar publicado por el Instituto Nacional de Estadística e Informática en el 2020 ^18^.

Se realizó un análisis univariado de frecuencias relativas y absolutas para describir las características de las escuelas, la oferta de bebidas y alimentos, la presencia de octógonos en las bebidas y alimentos, y la publicidad en los quioscos y cafeterías escolares como en los vendedores ambulantes. Para este análisis se empleó el programa estadístico Stata versión 17.

### Aspectos éticos

El protocolo del estudio fue exonerado de revisión por el Comité Institucional de Ética de la Universidad Peruana Cayetano Heredia, mediante la constancia CIEI-321-30-23.

## RESULTADOS

Se analizó la información de 57 quioscos y cafeterías escolares correspondiente a 45 escuelas, ya que 9 de ellas contaban con más de un quiosco o cafetería. Las 45 escuelas están distribuidas en 20 distritos de distintas zonas de Lima Metropolitana: el 37,8% en el sur, el 24,4% en el centro, el 20,0% en el norte y el 17,8% en el este. El 66,7% de escuelas son de gestión pública, y el 68,9% se ubican en zonas de nivel socioeconómico medio y medio bajo. La mediana de alumnos por escuela es de 1088 (Rango intercuartílico: 461 - 1451), y más de la mitad de las escuelas (55,6%) dispone de los tres niveles educativos (inicial, primaria y secundaria). En el 91,1% de escuelas, se encontraron vendedores ambulantes de bebidas y alimentos en la puerta de salida de los alumnos o en la acera de enfrente (ver detalle en la Tabla 2).

**Tabla 2:** Características de las escuelas de Lima Metropolitana incluidas en el análisis (n=45).

Características		n	%
Tipo de gestión			
	Pública	30	66,7
	Privada	15	33,3
Niveles educativos*			
	Primaria	2	4,4
	Secundaria	3	6,7
	Inicial y primaria	5	11,1
	Primaria y secundaria	10	22,2
	Inicial, primaria y secundaria	25	55,6
Ubicación			
	Lima centro	11	24,4
	Lima norte	9	20,0
	Lima este	8	17,8
	Lima sur	17	37,8
Nivel socioeconómico**			
	Alto	5	11,1
	Medio alto	5	11,1
	Medio	17	37,8
	Medio bajo	14	31,1
	Bajo	4	8,9
Mediana de número de alumnos*		1088 (461 - 1451)	
Mediana de número de alumnos según cantidad de niveles educativos*			
	Un nivel	350 (322 - 448)	
	Dos niveles	1146 (622 - 1387)	
	Tres niveles	1175 (781 - 1488)	
Número de vendedores ambulantes en la puerta de salida			
	Ningún vendedor ambulante	4	8,9
	1 vendedor ambulante	6	13,3
	2 vendedores ambulantes	7	15,6
	3 vendedores ambulantes	9	20,0
	4 vendedores ambulantes	6	13,3
	5 vendedores ambulantes	6	13,3
	6 vendedores ambulantes	3	6,7
	7 vendedores ambulantes	3	6,7
	8 vendedores ambulantes	1	2,2
Número de quioscos por institución educativa			
	1 quiosco o cafetería	36	80
	2 quioscos o cafetería	6	13,3
	3 quioscos o cafetería	3	6,7

*Información basada en la Estadística de la Calidad Educativa (ESCALE) del Ministerio de Educación del Perú. **El nivel socioeconómico se basa en los Planos estratificados de Lima Metropolitana a nivel de manzanas 2020 según ingreso per cápita del hogar del Instituto Nacional de Estadística e Informática (INEI).

### Oferta de bebidas y alimentos en quioscos y cafeterías escolares

En cuanto a la oferta de bebidas y alimentos, De los 57 quioscos y cafeterías escolares incluidas en el análisis, en el 96,5% se observaron productos procesados y ultraprocesados, y en el 89,5% se observó al menos un producto con octógono. Las categorías de productos procesados y ultraprocesados halladas con mayor frecuencia fueron los productos de panadería (96,5%), las bebidas industrializadas (89,5%), las golosinas (86,0%) y los snacks salados y dulces (84,2%). Del total de quioscos y cafeterías escolares que ofertaban productos de panadería, se observó al menos un producto con octógono en el 80,0%; de aquellos con snacks salados y dulces, en el 79,2%; de los que ofertaban golosinas, en el 69,4%; y de los que ofertaban bebidas industrializadas, en el 58,8%. La proporción de quioscos y cafeterías que ofertaban productos con octógonos de otras categorías puede consultarse en la Tabla 3.

**Tabla 3:** Oferta de bebidas y alimentos en quioscos y cafeterías escolares y en vendedores ambulantes en la puerta de las 45 escuelas de Lima Metropolitana.

Características			Quioscos y cafeterías escolares (n=57)		Vendedores ambulantes en la puerta de la escuela (n=148)	
			n	%	n	%
Oferta de bebidas y alimentos procesados y ultraprocesados						
	Productos de panadería		55	96,5	75	50,7
		con octógonos	44	80,0	49	65,3
	Golosinas		49	86,0	64	43,2
		con octógonos	34	69,4	35	54,7
	Snacks salados y dulces		48	84,2	66	44,6
		con octógonos	38	79,2	49	74,2
	Frutos secos envasados		16	28,1	8	5,4
		con octógonos	6	37,5	5	62,5
	Bebidas		51	89,5	62	41,9
		con octógonos	30	58,8	22	35,5
	Helados		16	28,1	29	19,6
		con octógonos	9	52,9	14	48,3
	Cereales		20	35,1	25	16,9
		con octógonos	13	65,0	22	88,0
	Yogurt		19	33,3	33	22,3
		con octógonos	8	42,1	9	27,3
	Sopas instantáneas		2	3,5	1	0,7
		con octógonos	2	100,0	1	100,0
	Otros (ingredientes)		2	3,5	1	0,7
		con octógonos	1	50,0	0	0,0
Oferta de bebidas y alimentos preparados						
	Preparaciones en base a embutidos		30	52,6	30	20,3
	Preparaciones de platos caseros		34	59,7	30	20,3
	Sanguches sin embutidos		32	56,1	19	12,8
	Bebidas naturales		32	56,1	33	22,3
	Preparaciones refrigeradas o congeladas		28	49,1	36	24,3
	Otras preparaciones		3	5,3	14	9,5
Oferta de alimentos naturales						
	Frutos secos a granel		14	24,6	8	5,4
	Frutas y verduras		29	50,9	21	14,2
Categorías de bebidas y alimentos procesados y ultraprocesados observadas						
	Ninguna categoría		2	3,5	33	22,3
	1 categoría		0	0,0	47	31,8
	2 categorías		2	3,5	6	4,1
	3 categorías		2	3,5	7	4,7
	4 categorías		17	29,8	19	12,8
	5 categorías		17	29,8	17	11,5
	6 categorías		10	17,5	13	8,8
	7 categorías		3	5,3	4	2,7
	8 categorías		2	3,5	1	0,7
	9 categorías		2	3,5	1	0,7
Categorías de bebidas y alimentos procesados y ultraprocesados con octógonos observadas						
	Ninguna categoría		6	10,5	76	51,4
	1 categoría		4	7,0	20	13,5
	2 categorías		7	12,3	12	8,1
	3 categorías		16	28,1	17	11,5
	4 categorías		13	22,8	10	6,8
	5 categorías		6	10,5	10	6,8
	6 categorías		2	3,5	1	0,7
	7 categorías		1	1,8	1	0,7
	8 categorías		0	0,0	1	0,7
	9 categorías		2	3,5	0	0,0

Por otro lado, el 86,0% de quioscos y cafeterías escolares ofertaban preparaciones caseras. Las categorías de preparaciones caseras observadas con mayor frecuencia son las preparaciones de platos caseros (59,7%), sanguches sin embutidos (56,1%) y las bebidas preparadas con insumos naturales (56,1%). Además, en el 61,4% de quioscos y cafeterías escolares se observó la venta de alimentos naturales, como frutos secos a granel (24,6%) y frutas y verduras (50,9%) (Tabla 3).

### Oferta de bebidas y alimentos en vendedores ambulantes

En las 41 escuelas con vendedores ambulantes, se observó un total de 148 vendedores. El 77,7% de ellos ofrecían bebidas y alimentos procesados y ultraprocesados, y en el 48,6% se observó al menos una bebida o alimento con octógono. Las categorías de productos procesados y ultraprocesados más observadas fueron productos de panadería (50,7%), snacks salados y dulces (44,6%), golosinas (43,2%) y bebidas industrializadas (41,9%). Del total de vendedores ambulantes que ofertaban snacks salados y dulces, en el 74,2% se observó al menos un producto con octógono; de los que ofertaban productos de panadería, en el 65,3%; de los que ofertaban golosinas, en el 54,7% y de los que ofertaban bebidas industrializadas, en el 35,5%. La proporción de vendedores ambulantes que ofertaban productos de otras categorías pueden consultarse en la Tabla 3. 

El 56,8% de los vendedores ambulantes ofertaban preparaciones caseras. Las preparaciones refrigeradas o congeladas (24,3%), las bebidas preparadas con insumos naturales (22,3%), las preparaciones de platos caseros (20,3%) y las preparaciones en base a embutidos (20,3%) fueron las categorías más observadas. Respecto a la oferta de alimentos naturales, se observó que el 17,6% de los vendedores ambulantes ofrecían este tipo de productos: en el 14,2% se observaron frutas y verduras, y en el 5,4% frutos secos a granel (Tabla 3). 

### Publicidad en quioscos o cafeterías escolares y en módulos ambulantes

El 57,9% de los quioscos y cafeterías escolares y el 27,7% de los vendedores ambulantes exhibían algún tipo de publicidad de bebidas y alimentos ultraprocesados. De los quioscos y cafeterías que exhibían publicidad, el 87,9% tenía la publicidad adherida al mobiliario y el 42,4% en forma de afiche o carteles. Respecto de los vendedores ambulantes que presentaban publicidad, el 95,1% la tenía adherida a sus módulos de venta, el 22% en forma de afiches o carteles, y el 4,9% en sus prendas de vestir. El número y tipo de publicidad por establecimiento se puede observar en la Tabla 4.

**Tabla 4:** Presencia y tipo de publicidad de bebidas y alimentos procesados y ultraprocesados en quioscos y cafeterías escolares y en los módulos de vendedores ambulantes en la puerta de 45 escuelas de Lima Metropolitana

Características		Quioscos y cafeterías escolares que presentan publicidad (n=33)		Módulos de vendedores ambulantes que presentan publicidad (n=41)	
		n	%	n	%
	Presentan publicidad	33	57,9	41	27,7
Tipo de publicidad*					
	Afiche	14	42,4	9	22,0
	Mobiliario	29	87,9	39	95,1
	Otro (prendas de vestir)	0	0,0	2	4,9
Número de establecimientos con publicidad adherida al mobiliario					
	1 mobiliario	8	27,6	22	56,4
	2 mobiliarios	7	24,1	16	41,0
	3 mobiliarios	9	31,0	0	0,0
	4 mobiliarios	2	6,9	0	0,0
	5 mobiliarios	1	3,4	0	0,0
	6 mobiliarios	2	6,9	1	2,6
Número de establecimientos con afiches o carteles publicitarios					
	1 afiche	3	21,4	4	44,4
	2 afiches	2	14,3	2	22,2
	3 afiches	5	35,7	0	0,0
	4 afiches	2	14,3	0	0,0
	5 afiches	2	14,3	1	11,1
	6 afiches	0	0,0	1	11,1
	7 afiches	0	0,0	1	11,1

* La suma de los valores supera el 100% ya que en algunos establecimientos se observó más de un tipo de publicidad.

## DISCUSIÓN

Los resultados muestran que casi la totalidad de los 57 quioscos y cafeterías de 45 escuelas públicas y privadas de Lima Metropolitana ofertan bebidas y alimentos procesados y ultraprocesados. Además, tras cuatro años de publicados los *Lineamientos* que prohíben la oferta de productos con octógonos en escuelas ^9^, 9 de cada 10 quioscos y cafeterías vendían productos procesados y ultraprocesados altos en azúcar, sodio o grasas saturadas, o con grasas trans. Sumado a esto, solo 6 de cada 10 quioscos y cafeterías ofertaban preparaciones caseras, refrescos naturales o sanguches sin embutidos, y solo la mitad de los quioscos y cafeterías ofertaban frutas y/o verduras. Esto evidencia que los escolares se encuentran expuestos a productos ultraprocesados en la escuela.

Los resultados son similares y en algunos casos más preocupantes que los encontrados en 2019, a cuatros meses de publicado los Lineamientos, cuando las 15 escuelas evaluadas ofertaban productos procesados y ultraprocesados, y el 73,3% ofrecían productos con octógonos ^13^, contra el 90% del estudio actual.

La prohibición del expendio de bebidas y alimentos no saludables en las escuelas es una estrategia efectiva en la lucha contra el incremento del sobrepeso infantil ^19^, por lo que diversos países de Latinoamérica, además del Perú, han implementado políticas que restringen la oferta de estos alimentos en escuelas. Lamentablemente, la deficiente implementación de las normativas que regulan la oferta de alimentos en las escuelas se ha observado también en otros países de la región. En México, a 8 años de publicada la directriz que prohíbe la venta de alimentos y bebidas que no cumplan con los criterios nutricionales establecidos en la misma, solo el 1% de los 103 quioscos escolares evaluados cumplió totalmente con la normativa ^(^^20^. En Ecuador, a nueve años de publicada su política que regula el expendio de alimentos no saludables en quioscos escolares, propietarios de quioscos, directores de escuelas y padres indicaron que no se cumple dicha política ^21^. De igual forma, en Costa Rica se evidenció que el 76,6% de alimentos expendidos en quioscos escolares no cumplían con los criterios nutricionales exigidos en la política que prohíbe la oferta de productos y preparaciones con alto contenido de grasas, azúcares totales y sodio en escuelas a nueve años de ser publicada ^22^.

En contraste con esta situación mayoritaria, en Chile se evidenció que la prohibición de la venta de bebidas y alimentos con octógonos en los quioscos escolares redujo la disponibilidad de estos productos del 90,4% al 15,0% a dos años de su publicación ^23^. La experiencia chilena revela que las políticas de alimentación escolar pueden ser efectivas si son eficientemente implementadas.

Además de la oferta en el interior de los colegios se observaron vendedores ambulantes en los exteriores de la enorme mayoría de escuelas evaluadas, lo que evidencia el incumplimiento de la ordenanza municipal del 2021 que restringe la venta ambulatoria en los 200 metros próximos a las escuelas de Lima Metropolitana ^16^. Además, la mitad de los vendedores ambulantes ofertaban bebidas y alimentos procesados y ultraprocesados con octógonos, lo que indica que, aunque las escuelas cumplieran con prohibir su venta, los escolares seguirán encontrando estos productos en la puerta de salida. En el 2019 se encontró una situación muy similar en relación con la presencia de vendedores ambulantes ^13^, lo cual sugiere que a pesar de la promulgación de la ordenanza no se han dado mejoras.

Cabe resaltar que las categorías de alimentos ofertadas con mayor frecuencia en los quioscos y en los vendedores ambulantes fueron los productos de panadería que incluyen las galletas y queques industrializados, los snacks, las golosinas o dulces y las bebidas industrializadas. Una situación similar se encontró en México, donde en más del 90% de quioscos escolares evaluados se hallaron productos de panadería, dulces y bebidas azucaradas como gaseosas, néctares y jugos ^20^. Esto muestra que, a pesar de la prohibición de la oferta de estos productos, existe una alta disponibilidad de los mismos, lo que, sumado su bajo precio, hace que la accesibilidad de los escolares a estos productos se incremente.

Sumado a la alta accesibilidad a bebidas y alimentos ultraprocesados se encontró publicidad de los mismos en 6 de cada 10 quioscos o cafeterías escolares, lo cual influye en las decisiones de compra de los escolares ^24^, más aún si esta se encuentra dentro de un espacio donde pasan varias horas del día como la escuela. Si bien el Perú no cuenta con una regulación que prohíba la publicidad en el entorno escolar, se exige que toda publicidad que presente alguna bebida o alimento con octógonos deben incluir este mismo en la esquina superior derecha de la publicidad. Basado en dicha exigencia, el mayor tipo de publicidad hallada en el presente estudio se encuentra adherida a los muebles como congeladoras, anaqueles, exhibidoras, entre otros, ya que este tipo de publicidad solo incluye la marca o nombre de bebidas y alimentos fácilmente identificables por los escolares, más no el producto, lo que significa que, con la actual regulación, no les corresponde cumplir con la exigencia de incluir los octógonos correspondientes en dicha publicidad.

Frente a la situación descrita en este estudio, es relevante reflexionar sobre los posibles motivos del incumplimiento de estas políticas, que podrían incluir la deficiente fiscalización por parte de las autoridades responsables ^20^^-^^22^, la falta de empoderamiento de la comunidad educativa para ejercer un rol fiscalizador y la ausencia de sanciones efectivas ante el incumplimiento de la política ^21^. Para su adecuada implementación, las políticas deben disponer explícitamente los métodos y los responsables de la fiscalización, quienes deberían presentar informes periódicos de sus visitas. Así también se deben detallar las sanciones correspondientes ante el incumplimiento de la política. Por otro lado, las autoridades responsables de la implementación de la política deben asegurar que los propietarios de los quioscos y cafeterías escolares como los encargados de la fiscalización reciban capacitación que les permita interpretar correctamente lo estipulado en la política ^(^^21^^,^^22^. Así como cada apartado de la política debe incluir términos previamente definidos para que asegure su correcta y fácil interpretación, por ejemplo, en la Ordenanza N° 2366-2021 establece una zona rígida para el comercio ambulatorio de alimentos no saludables, sin embargo, no se define que alimentos son considerados no saludables lo que puede generar confusiones en los expendedores y en consecuencia incumplir la política.

Otro posible motivo de la situación encontrada se relaciona con las preferencias alimentarias de los escolares. Al respecto, estudios cualitativos realizados a propietarios de quioscos escolares reportan que los alimentos saludables son poco atractivos para esta población ^21^, lo que puede deberse tanto a la deficiente educación en alimentación saludable como a la constante exposición a publicidad de alimentos ultraprocesados en distintos medios ^20^^-^^22^. Además, la presencia de vendedores ambulantes profundizan esta problemática, ya que los escolares buscarían conseguir alimentos poco saludables antes o después de clases en el exterior del colegio ^20^^-^^22^. Sumado a ello se ha identificado que en escolares de escuelas de bajos ingresos la presencia del octógono en los alimentos y bebidas ultraprocesados parece no ser suficiente para la elección de alimentos más saludables ^25^. Además, muchos de los alimentos procesados y ultraprocesados expendidos en las escuelas generalmente se presentan en empaques de tamaño pequeño (cara frontal menor a 50 cm^2^), los cuales, según la política establecida en Perú, pueden están exentos de llevar octógonos a pesar de tener un alto contenido de uno o más nutrientes regulados, esto puede generar confusiones, ya que los escolares pueden asumir un alimento como saludable cuando puede no serlo ^26^. Ante ello, es sumamente relevante, en primer lugar, establecer un programa educativo en alimentación saludable dirigido tanto a los escolares como a sus familias y sus docentes con el fin de sensibilizar en las posibles consecuencias de consumir alimentos poco saludables. En segundo lugar, se debe hacer efectiva la fiscalización tanto en quioscos y cafeterías escolares como de vendedores ambulantes que ofrecen alimentos poco saludables alrededor de las escuelas. En tercer lugar, realizar campañas publicitarias de alimentos saludables equiparables con las que existen para alimentos poco saludables, además de generar políticas que prohíban cualquier tipo de publicidad de bebidas y alimentos procesados, y ultraprocesados tanto en el interior como en el entorno próximo a las escuelas, y así contribuir en la implementación de ambientes alimentarios escolares saludables.

Finalmente, también existirían motivos económicos, ente los cuales se encuentra el bajo poder adquisitivo de los escolares ^(^^20^^,^^22^ lo que nos les permite adquirir alimentos saludables que suelen tener precios más elevados que los alimentos procesados y ultraprocesados ^27^. Además, por parte de los propietarios de los quioscos, la necesidad de cubrir gastos propios de su actividad comercial como el costo de alquiler, personal y servicios básicos es su principal barrera para variar la oferta de alimentos, ya que como la oferta de alimentos saludables es poco atractiva para los escolares, no es demandada de forma similar que los productos poco saludables, lo que se traduce en una disminución en sus ingresos y por ende una baja rentabilidad ^21^^,^^22^.

La oferta y publicidad de productos ultraprocesados en quioscos y cafeterías escolares, así como en los entornos de las escuelas, puede favorecer la adopción de hábitos alimentarios no saludables en niños, niñas y adolescentes. Para enfrentar este problema, es necesario que las autoridades en salud brinden herramientas a los propietarios de los quioscos y cafeterías escolares que les permitan ofrecer alimentos saludables, atractivos y accesibles, asegurando al mismo tiempo su rentabilidad. Una medida concreta es la elaboración de listas de bebidas, alimentos y preparaciones recomendadas, adaptadas a las distintas temporadas del año e incorporando insumos locales. Otra estrategia consiste en implementar subsidios a los alimentos saludables y aplicar impuestos a los productos no saludables, siguiendo el ejemplo de las bebidas azucaradas, cuya regulación ha demostrado efectos beneficiosos en la salud ^28^^,^^29^. Estas políticas pueden incentivar a los propietarios a ofrecer con mayor frecuencia opciones saludables y rentables. Finalmente, resulta indispensable revisar y modificar la normativa vigente, ya que persisten vacíos regulatorios como la falta de restricciones publicitarias en el entorno escolar, la ausencia de precisión sobre el uso obligatorio del etiquetado frontal de advertencia en avisos publicitarios que muestran la marca, pero no el producto, y la carencia de una definición clara de alimentos no saludables en la Ordenanza Municipal N° 2366-2021. En conjunto, estas acciones permitirían fortalecer el entorno escolar como un espacio que promueva efectivamente la alimentación saludable.

Una fortaleza del estudio es que se contó con docentes o madres de familia como observadores, quienes registraron la oferta y publicidad a la que cotidianamente están expuestos los escolares dentro de la escuela. Esto hubiera resultado complicado para personas ajenas a la institución, cuya presencia podría inducir a un cambio en la oferta de bebidas y alimentos.

Entre las limitaciones del estudio, se encuentra la falta de representatividad de la muestra, que limita la generalización de los resultados. No obstante, la inclusión de casi 50 escuelas públicas y privadas, de diferentes zonas y niveles socioeconómicos, ofrece valiosos resultados sobre la oferta de bebidas y alimentos, y el cumplimiento de la normativa actual. Otra limitación es la heterogeneidad de los observadores, sin embargo, esto se pudo controlar mediante una capacitación personalizada y minuciosa en el registro de datos en el instrumento. Así también, se considera como la limitación que las observaciones se realizaron en una única fecha, por lo que no se pudo capturar la posible variación en la oferta de bebidas y alimentos. Finalmente, las observaciones sólo registraron la presencia de bebidas y alimentos por categorías, sin determinar la cantidad o diversidad de productos ofertados en cada categoría ni las ventas, lo que daría más información sobre los alimentos ofertados.

En conclusión, en casi la totalidad de quioscos y cafeterías escolares observados se ofertan bebidas y alimentos procesados y ultraprocesados, y en 9 de cada 10, se ofrecen bebidas y alimentos con octógonos, mientras solo en la mitad se ofertan frutas y/o verduras. En 6 de cada 10 quioscos y cafeterías se observó publicidad de bebidas y alimentos procesados, y ultraprocesados. Además, la venta ambulatoria de bebidas y alimentos procesados, y ultraprocesados con octógonos se observa en más de la mitad de las escuelas. Esta situación refleja que los niños, niñas y adolescentes están expuestos a entornos alimentarios escolares no saludables a pesar de contar con normativas vigentes que regulan esta temática. Resulta fundamental que las autoridades responsables de la implementación de las políticas fortalezcan y garanticen el monitoreo y la vigilancia del cumplimiento de las mismas para asegurar así su efectividad, y alcanzar sus objetivos de mejorar la salud pública de niños, niñas y adolescentes.
